# Histone Modifications within the Human X Centromere Region

**DOI:** 10.1371/journal.pone.0006602

**Published:** 2009-08-12

**Authors:** Brankica Mravinac, Lori L. Sullivan, Jason W. Reeves, Christopher M. Yan, Kristen S. Kopf, Christine J. Farr, Mary G. Schueler, Beth A. Sullivan

**Affiliations:** 1 Institute for Genome Sciences & Policy, Duke University, Durham, North Carolina, United States of America; 2 University Program in Genetics and Genomics, Duke University, Durham, North Carolina, United States of America; 3 Department of Genetics, University of Cambridge, Cambridge, United Kingdom; 4 National Human Genome Research Institute, National Institutes of Health, Bethesda, Maryland, United States of America; 5 Department of Molecular Genetics and Microbiology, Duke University Medical Center, Durham, North Carolina, United States of America; Texas A&M University, United States of America

## Abstract

Human centromeres are multi-megabase regions of highly ordered arrays of alpha satellite DNA that are separated from chromosome arms by unordered alpha satellite monomers and other repetitive elements. Complexities in assembling such large repetitive regions have limited detailed studies of centromeric chromatin organization. However, a genomic map of the human X centromere has provided new opportunities to explore genomic architecture of a complex locus. We used ChIP to examine the distribution of modified histones within centromere regions of multiple X chromosomes. Methylation of H3 at lysine 4 coincided with DXZ1 higher order alpha satellite, the site of CENP-A localization. Heterochromatic histone modifications were distributed across the 400–500 kb pericentromeric regions. The large arrays of alpha satellite and gamma satellite DNA were enriched for both euchromatic and heterochromatic modifications, implying that some pericentromeric repeats have multiple chromatin characteristics. Partial truncation of the X centromere resulted in reduction in the size of the CENP-A/Cenp-A domain and increased heterochromatic modifications in the flanking pericentromere. Although the deletion removed ∼1/3 of centromeric DNA, the ratio of CENP-A to alpha satellite array size was maintained in the same proportion, suggesting that a limited, but defined linear region of the centromeric DNA is necessary for kinetochore assembly. Our results indicate that the human X centromere contains multiple types of chromatin, is organized similarly to smaller eukaryotic centromeres, and responds to structural changes by expanding or contracting domains.

## Introduction

The centromere is a crucial locus for maintaining genome stability. It is the foundation for kinetochore formation, and directs the proper chromosomal segregation during cell division. Improper assembly or function at centromeres is responsible for cell cycle defects and genome instability [1], [2], [3]. Although centromeres are essential loci that are functionally similar, they show little consistency in DNA sequence content, ranging from the sequence-dependent 125 bp point centromere in the budding yeast *Saccharomyces cerevisiae* to multi-megabase, epigenetically-regulated regional centromeres in primates [4]. The various roles of non-coding sequences, such as non-coding RNAs, microRNAs and siRNAs, in genome organization and regulation emphasize the importance in understanding how large megabase-sized regions of the DNA ensure genome stability and chromosome inheritance in meiosis and mitosis [5].

Replacement of core histones with histone variants, as well as posttranslational, covalent modification (acetylation, phosphorylation, methylation and ubiquitination) of the amino-terminal tails of histones correlate with distinctive chromatin states, such as transcriptionally repressive heterochromatin and open euchromatin that supports transcription [6]. Centromeres contain the histone H3 variant, CENP-A that replaces core H3 within centromeric nucleosomes. Not all H3 is replaced by CENP-A, and in fact, centromeric chromatin contains alternating subdomains of CENP-A and H3-containing nucleosomes [7], where H3 is dimethylated at lysine residue 4 (H3K4me2). H3K4me2, a modification associated predominantly with poised euchromatin, distinguishes CEN chromatin from surrounding blocks of chromatin that are enriched for H3K9me2 and H3K9me3 nucleosomes [8], [9]. A similar model for centromere organization is present in other organisms, such as fission yeast and *Drosophila* [9], [10].

**Figure 1:**
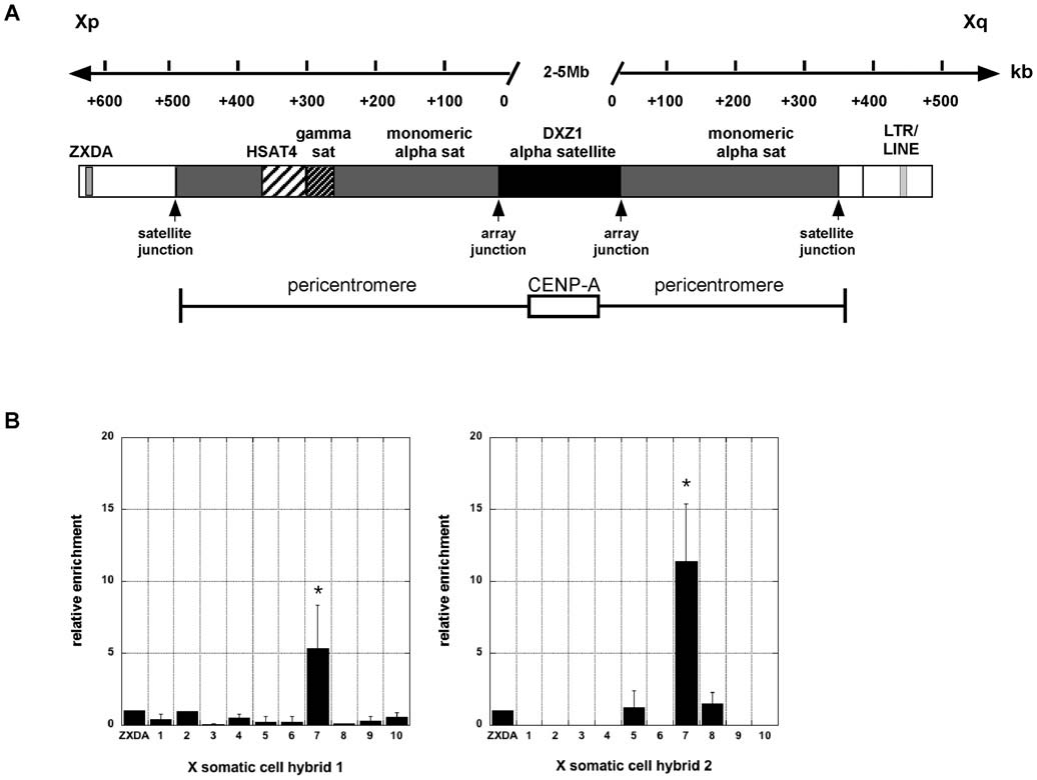
Overview of human X centromere region. A. The short (Xp) and long (Xq) arm sides of the centromere are designated. The solid black bar represents higher order repeat (HOR) DXZ1 alpha satellite DNA which is composed of highly ordered 171 bp monomers organized into multi-megabased arrays. The array is not drawn to scale and is shown as the point from which the other pericentromeric genomic positions are centered. Each type of pericentromeric DNA is designated by differently shaded boxes. Regions of unordered alpha satellite monomers flank HOR alpha satellite. The short arm pericentromere contains other types of satellites such as gamma satellite and HSAT4. The junctions between DXZ1 alpha satellite (array junction) and between monomeric satellite and the chromosome arms (satellite junction) are denoted by arrowheads. The two sites located 150 kb outside of the centromere, ZXDA and LTR/LINE, are depicted because they were included in the ChIP analyses. The location of chromatin domains for CENP-A chromatin (kinetochore) and the flanking pericentromere (heterochromatin) are shown relative to the schematic genomic region. B. ChIP analysis using antibodies specific for mouse Cenp-A were used to confirm that mouse Cenp-A replaces human CENP-A at higher order alpha satellite DNA on the X chromosome that was transferred into a mouse cell line. ZXDA was used for normalization and establishment of background levels of enrichment (n = 3 with SD). Asterisks indicate statistically significant differences as calculated by a Student's t-test (*p*<0.05).

The major DNA element of human centromeres is alpha satellite, a 171 bp repeat that is tandemly organized either as multimeric, higher–order repeat (HOR) arrays or as heterogeneous monomers lacking periodicity or hierarchy (monomeric alpha satellite) [11]. Centromeres are composed entirely of repetitive elements that have restricted accessibility to sequencing and assembling. Human genome assemblies end before extending through pericentromeric satellite DNA to the chromosome-specific HOR alpha satellite arrays. The X chromosome was the first chromosome for which a sequence assembly spanning both sides of the pericentromere was achieved [12], [13]. These *tour de force* studies revealed that the X pericentromere is a complex mix of satellite families and transposable elements distributed between the euchromatic arm sequences and the homogenous array of chromosome-specific HOR alpha satellite DNA [13], [14]. The X chromosome-specific HOR array (DXZ1) comprises approximately 2% of the entire chromosome length, although its size is heterogeneous, ranging 1.5–5 Mb, between homologues and among individuals [15]. DXZ1 not only genetically defines the centromere, but functionally is the site of kinetochore assembly (Fig. 1A) [13]. The kinetochore, marked by centromere proteins, such as CENP-A and CENP-C, is only assembled on a portion of the multi-megabase DXZ1 array [8], [16]. The pericentromere region is comprised of divergent alpha satellite and other repetitive sequences (Fig. 1A). Monomeric alpha satellite arrays span 500 kb of Xp pericentromere and 350 kb of Xq pericentromere [12], [13]. The Xp pericentromeric region also contains additional repetitive DNA, such as gamma satellite, human satellite 4 (HSAT4), and transposable elements such as LINE repeats [13], [14]. Abrupt sequence transitions separate the various satellite repeats, including monomeric and HOR alpha-satellite (array junction), as well as euchromatic arms and monomeric arrays (satellite junctions) [17]. In fission yeast, the CENP-A^Cnp1^ chromatin core and heterochromatic pericentromeric repeats are separated by a defined sequence boundary [18]. In this organism, the flanking repeats are important for chromosome stability [19], [20]. In humans, however, systematic deletion of X pericentromeric sequences affects chromosome segregation and mitotic stability only slightly [21], [22]. Epigenetic and functional characteristics of large pericentromeric regions in humans that separate the kinetochore region from the chromosome arms are not well defined.

In this work, we define a profile of histone modifications at various locations across the human X centromeric genome assembly. Chromatin immunoprecipitation-PCR (ChIP-PCR) with antibodies against various methylated lysine residues within histone tails was used to study heterochromatin and euchromatin enrichment across a ∼5 Mb region. We interrogated 10 genomic sites, including transitions between different satellite arrays and between satellite arrays and chromosome arms, across multiple X centromeres. We also explored preservation of histone modification patterns at centromeres of human X chromosomes that had been transferred into interspecies hybrids. To evaluate the relationship between chromosome stability and long-range chromatin organization, we compared histone modification profiles at an X centromere before and after structural rearrangement. We have compiled a broad view of heterochromatin and euchromatin across a highly repetitive human centromere region.

## Results

### Chromosome-specific markers located within X centromere and pericentromere region

To identify X centromere-specific markers suitable for ChIP, genomic sequence spanning 450 kb of Xp pericentromere, 350 kb of Xq pericentromere, and regions 150 kb from Xp and Xq centromere-arm junction was analyzed *in silico* using UCSC Genome Browser (March 2006) human reference sequence assembly (NCBI Build 36.1). Specific primer sets were designed to amplify major genomic boundaries, such as centromere-arm (sat jxn) and monomeric-HOR array junctions (array jxn) [12], [13], as well as between and within blocks of various satellite DNAs located within the pericentromere (Fig. 1A). Two sites, each located 150 kb outside of the centromere, one in Xp (ZXDA) and one in Xq (site 9), were non-centromeric assay points. Since these sites are located outside the centromere, we expected to observe a shift from centromeric histone modifications to those more indicative of euchromatin.

### Euchromatic modifications are largely excluded from the extended centromere

**Figure 2:**
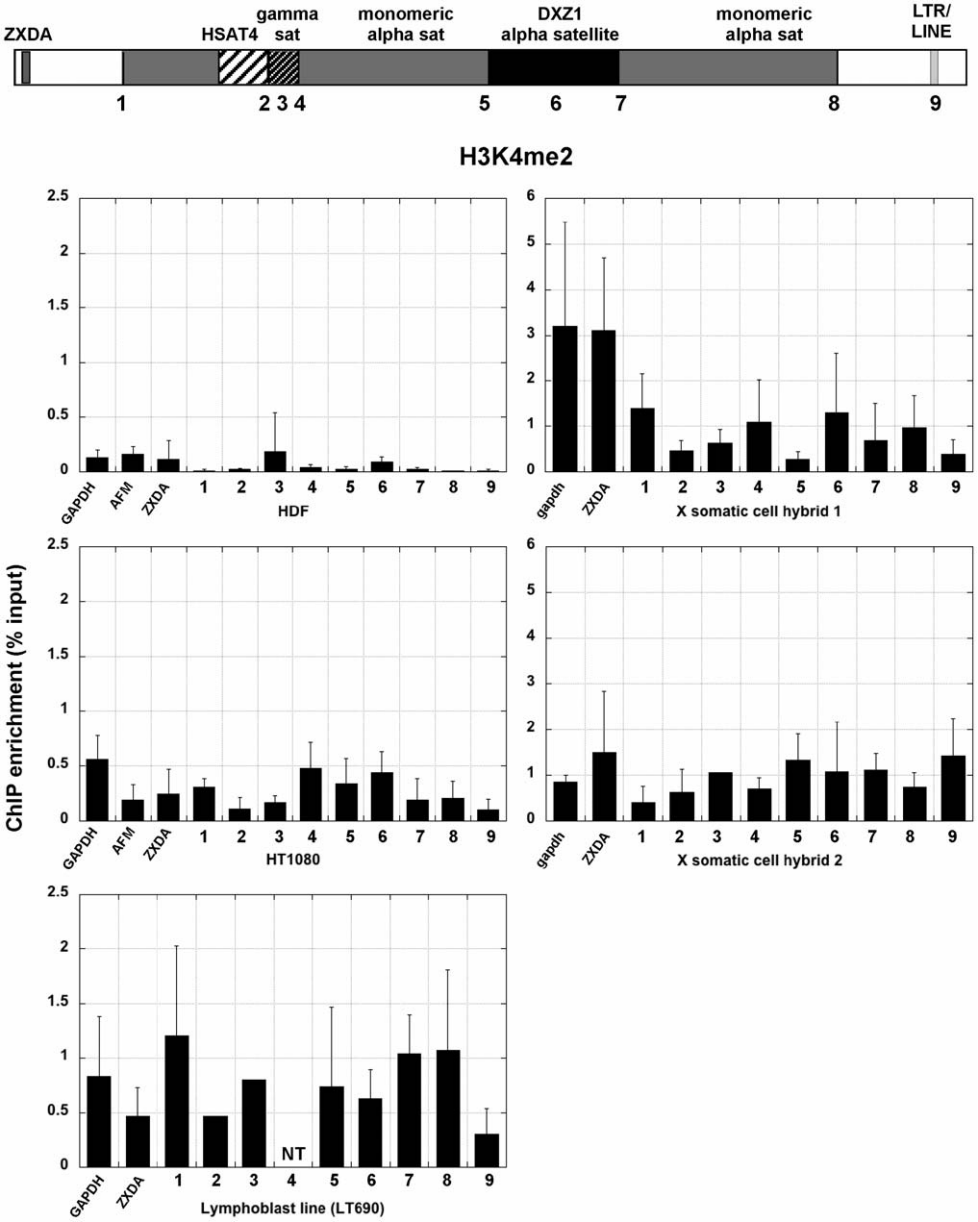
H3K4me2 across the X centromere in primary and immortalized human cells and two mouse-human hybrids containing X as the only human chromosome. The schematic at the top of the figure shows the structure of the X centromere. Each number along the centromere represents a genomic site that was interrogated by ChIP-PCR with a specific histone antibody. Each bar graph shows relative enrichment for each histone modification (n≥3 with standard deviation, SD). Control regions, including GAPDH, AFM and X-linked ZXDA are also included. The bar graph shows enrichment calculated as percentage of input.

Native chromatin was prepared from isolated nuclei from several different human cell lines (Table 1) and digested with micrococcal nuclease to generate fragments less than 1000 bp (<7 nucleosomes). This digested chromatin was used in ChIP with a panel of antibodies specific to methylated forms of H3K4, H3K9, H3K27 and H4K20. Precipitated DNA was analyzed by semi-quantitative PCR with primer sets spanning the X pericentromere/centromere region (Fig. 2; Table 2). With the exception of the primers for DXZ1, all primer pairs amplified genomic fragments less than 500 bp. At least three independent ChIP experiments were performed on each cell line, and PCRs were done in duplicate for every modification in each experiment. Enrichment for each modification at a specific site was calculated as a percentage of the input. Control genes, such as GAPDH and AFM, were included in the analyses. A primer pair 5′ to ZXDA, the first proximal gene located 150 kb from the Xp centromere-arm boundary, was also included in the analyses. Primers corresponding to 150 kb from the Xq centromere-arm boundary were also included (site 9) to address symmetry of the pattern of modifications and to assess chromatin environment outside the centromere.

**Table 1:** Cell lines included in the study.

Cell line	Cell Type	Source/Reference	Karyotype/Notes
human dermal fibroblast (HDF)	primary neonatal foreskin fibroblasts	ATCC	46,XY
HT1080	fibrosarcoma	ATCC	46,XY/92,XXYY
LT690	lymphoblastoid	Ref. 15	46,XY
t60-12 (somatic cell hybrid 1)	mouse-human hybrid; L cell derived	Ref.42	contains single human active X chromosomes
Aha-11aB1 (somatic cell hybrid 2)	mouse-human hybrid; L cell derived	Ref. 42	contains single human active X chromosome
HTM18TC8	hamster-human hybrid	Refs. 21, 22	contains single human X chromosome
FA3Wg8-4	hamster-human hybrid	Refs. 21, 22	truncated X chromosome from HTM18TC8 line

**Table 2:** Primer sets used for ChIP-PCR.

Genomic site	primer name	description	length	Position Assembly March 2006	T_a_
**ZXDA**	BS254/255	F: 5′ TCAATTAAGGTGGGAGGCAG 3′ R: TGTGAGGTAATTATGGCAAAGTC 3′	217 bp	chrX:57949717-57949933	59°C
**1 (Xp sat jxn)**	BS236/237	F: 5′ ATTTTCCCAGCACCATTTTTCAA 3′ R: 5′ CTGTCAAGATGGTATGGGCTGTGT 3′	397 bp	chrX:58104524-58104920	56°C
**2 (HSAT4)**	BS392/393	F: 5′ TGTGGTCAGCGAGATGTCTC 3′ R: 5′ AGTTTCCTGTGTGACCCCAG 3′	159 bp	chrX:58259011-58259169	64°C
**3 (HSAT4-γ jxn)**	BS222/223	F: 5′ CCAGGCATTCAAGCGGGAGAG 3′ R: 5′ CGGCGGAAGTTATCGTTGAGAG 3′	355 bp	chrX:58296350-58296704	60°C
**4 (γ-satellite)**	BS160/161	F: 5′ TTC AAC GTA CCC CTG AAA GCC TGG 3′ R: 5′ CTA TTT TGT CCC AAG CCT GCC 3′	339 bp	chrX:58329603-58329941	58°C
**5 (γ-ALR jxn)**	BS226/227	F: 5′ AGCCCGAGGAAAATACTGGTGAGG 3′ R: 5′ GCTGTCTTTCTAGTTTTTGTCGTGGGTTAT 3′	224 bp	chrX:58335817-58336040	62°C
**6 (Xp mono-HOR jxn)**	BS240/241	F: 5′ AACGCTGCGCTATCAAAGGGAAAGT 3′ R: 5′ GGACATGTGGAGCGCTTTGTGC 3′	313 bp	chrX:58577182-58577494	64°C
**7 (α-satellite)**	BS13/14	F: 5′ ATAATTTCCCATAACTAAACACA 3′ R: 5′ TGTGAAGATAAAGGAAAAGGCTT 3′	535 bp	chrX:61610033-61610567	55°C
**8 (Xq HOR-mono jxn)**	BS258/259	F: 5′ GACCTCAAAGCACTCTAAATACAC 3′ R: 5′ CTTCACATAAAAACTAGACAGACAG 3′	495 bp	chrX:61642633-61643127	61°C
**9 (Xq sat jxn)**	BS238/239	F: 5′ CCTGCTGAATCAAAACAATGGT 3′ R: 5′ CAAAGAAGGCTGGGTGAGAAG 3′	389 bp	chrX:61962313-61962701	59°C
**10 (Xq jxn+150 kb)**	BS260/261	F: 5′ TGCCTCCATGATTCAGTTACCA 3′ R: 5′ AATCTCCCTCCTCTTACCCTCTA 3′	475 bp	chrX:62111658-62112132	58°C

Within the three human cell lines studied, the euchromatin-associated modification H3K4me2 was present at DXZ1 alpha satellite repeats (Fig. 2,), a result that is consistent with existing models showing that nucleosomes containing H3K4me2 are interspersed with CENP-A-nucleosomes at centromeres [8], [9], [10], [23]. In primary human dermal fibroblasts (HDF), H3K4me2 was detectable mainly at DXZ1 alpha satellite DNA. However, this modification was observed at several sites in the pericentromere in the transformed cell lines, HT1080 and a lymphoblast (EBV-transformed) line LT690 [24](Fig. 2). It was also decreased outside the centromere at site 9. Surprisingly, on X chromosomes that had been transferred into mouse cells, although CENP-A was concentrated on DXZ1 (Fig. 1B), H3K4me2 was not significantly enriched above the control genes, although it was not completely absent. In somatic cell hybrid line 2, H3K4me2 was also slightly enriched on gamma satellite DNA and HSAT4. Enrichment for H3K4me2 at multiple sites across the centromere in the transformed human cells compared to primary cells suggests that immortalization may cause notable chromatin changes at the human X centromere.

### Heterochromatic histone modifications are variably spread through X centromeres

**Figure 3:**
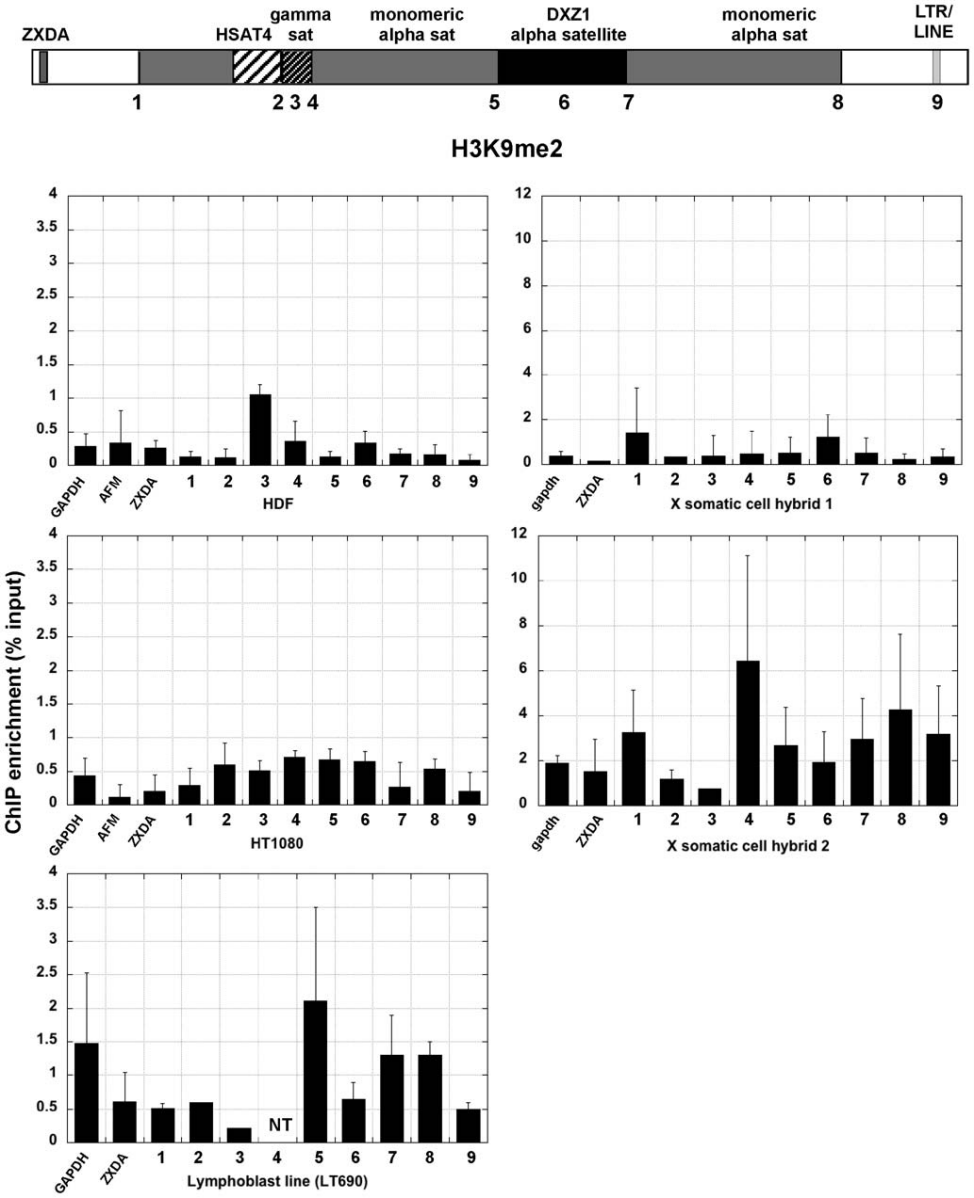
H3K9me2 enrichment at X centromeres in human cell lines and mouse-human hybrids. The schematic shows the structure of the X centromere. Each number along the centromere represents a genomic site or control region that was interrogated by ChIP-PCR with a specific histone antibody. Each bar graph shows relative enrichment for each histone modification (n≥3 with SD). Control regions, including GAPDH, AFM and X-linked ZXDA are also included. The bar graph shows enrichment calculated as percentage of input.

**Figure 4:**
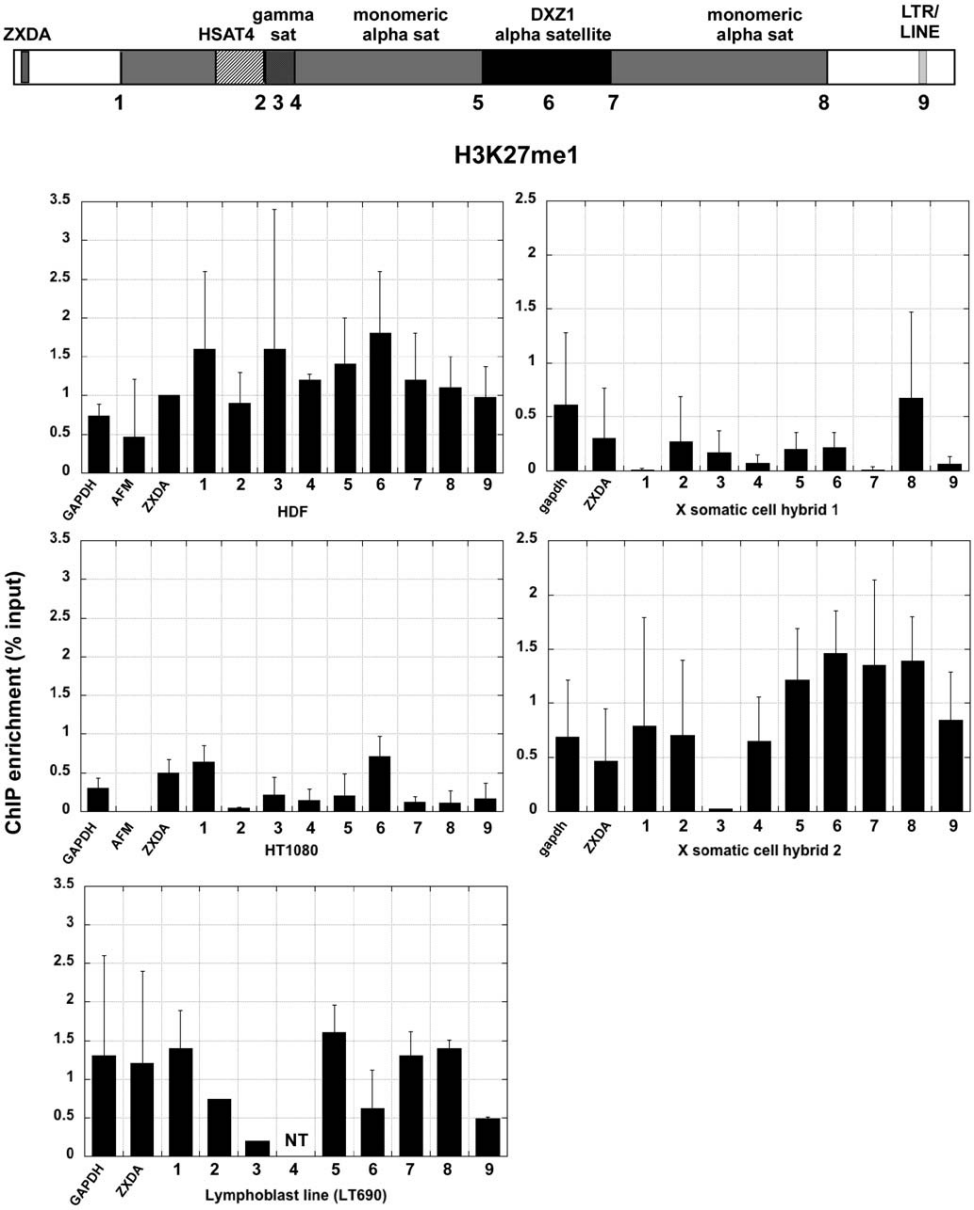
H3K27 mono-methylation (H3K27me1) at the X centromere in human cells and mouse-human somatic cell hybrids. Each number across the schematic representation of the centromere is a genomic site that was interrogated by ChIP-PCR. Control regions, including GAPDH, AFM and X-linked ZXDA are also included. The bar graph shows relative enrichment for H3K27 methylation (n = 3 with SD) calculated as percentage of input.

**Figure 5:**
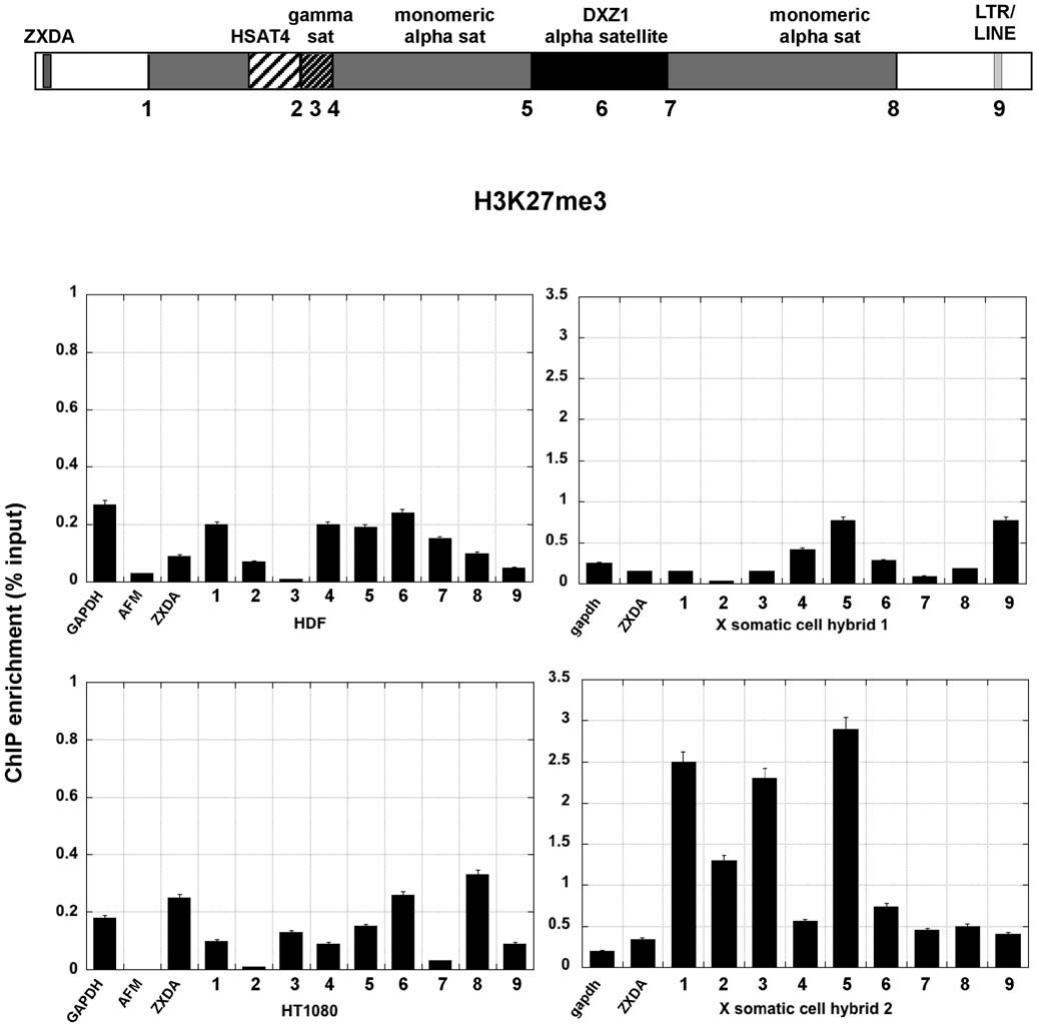
H3K27 trimethylation (H3K27me3) at the X centromere in human cells and mouse-human somatic cell hybrids. Each number across the schematic representation of the centromere is a genomic site that was interrogated by ChIP-PCR. The bar graph shows relative enrichment for H3K27 methylation (n = 3 with SD). Control regions, including GAPDH, AFM and X-linked ZXDA are also included. The bar graph shows relative enrichment for H3K27 methylation (n = 3 with SD) calculated as percentage of input.

**Figure 6:**
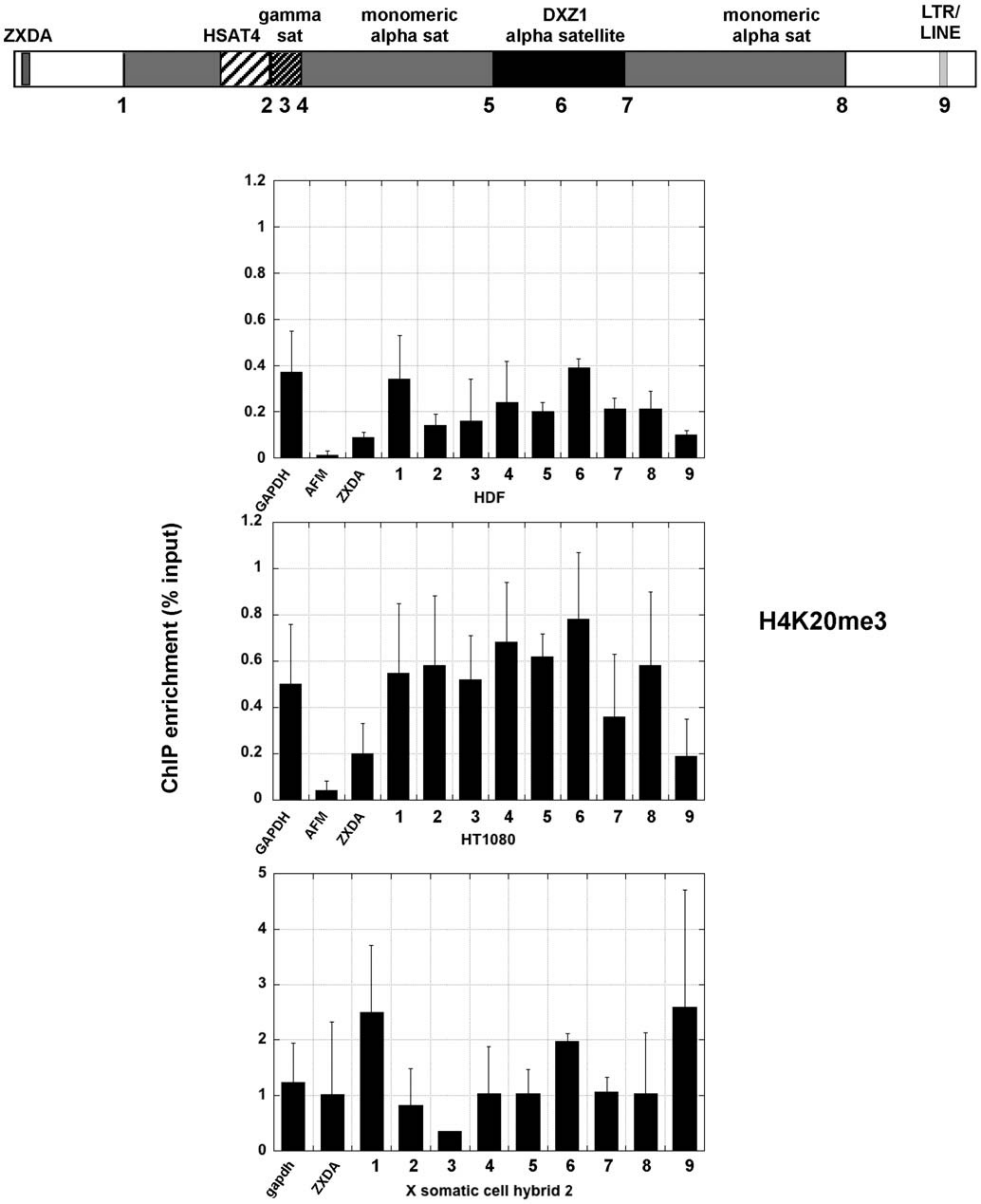
H4K20 trimethylation (H4K20me3) at the X centromere in two human cell lines and one of the mouse-human somatic cell hybrids. Each number across the schematic representation of the centromere is a genomic site that was interrogated by ChIP-PCR. Control regions, including GAPDH, AFM and X-linked ZXDA are also included. The bar graph shows relative enrichment for H3K27 methylation (n = 3 with SD) calculated as percentage of input.

Centromere regions have been historically associated with heterochromatin [25]. Heterochromatic histone modifications such as H3K9 methylation and H3K27 methylation, characterize yeast, plant, insect and mammalian centromeres, primarily at sequences flanking the CENP-A chromatin core [8], [9], [26], [27], [28]. In the human cell lines, H3K9me2 was enriched at DXZ1 alpha satellite and a few sites surrounding DXZ1 (sites 3, 4, 5 and 8) (Fig. 3). These results confirm that the portion of alpha satellite DNA not assembled into CENP-A:H3K4me2 chromatin is heterochromatic [8]. In the human cell lines H3K9me2 appeared to decrease at sites outside the centromere, particularly ZXDA and site 9 (Fig. 3)). On X centromeres transferred into somatic cell hybrids, H3K9 methylation was not highly enriched at any one site, but was more enriched than the control genes at most sites except site 3 (gamma satellite). The X centromere in somatic cell hybrid 2 also appeared to be much more heterochromatic than in somatic cell hybrid 1. Despite using multiple sources of H3K9me2 antibodies, hybrid 1 never showed great enrichment for H3K9 methylation. H3K27 methylation, was detected either at DXZ1 or flanking DXZ1 in the human cell lines and somatic cell hybrid 2 (Figs. 4, 5). Heterochromatin marked by H4K20me3 was also present at most sites in the pericentromere and at DXZ1 (Fig. 6). It appeared to decrease outside the pericentromere region, particularly in HDF and HT1080 lines. Our results indicate that the pericentromeric region between the centromere core and arms is largely heterochromatic.

### Structural alteration of the X centromere correlates with chromatin reorganization

**Figure 7:**
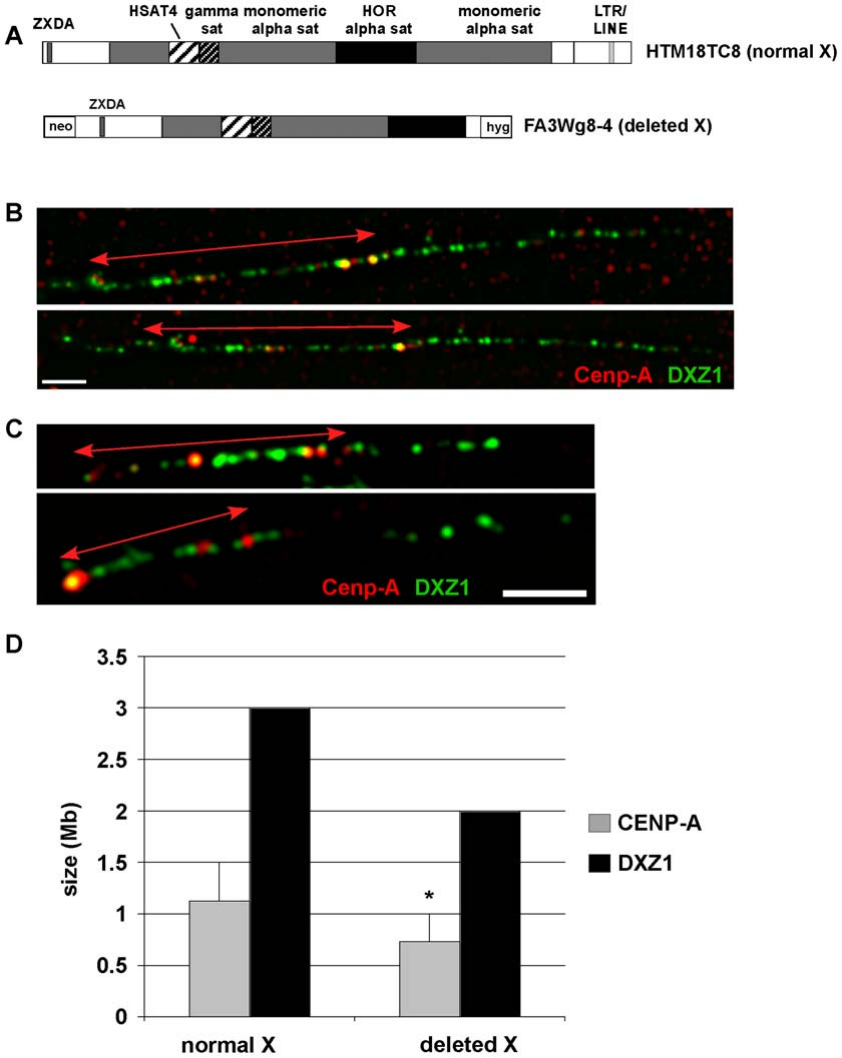
CENP-A mapping on X alpha satellite (DXZ1) on normal and partially deleted X chromosomes in a human-hamster somatic cell hybrid. *(A)* Schematic of the genomic structure of the X centromeres s of the normal X chromosome in the human:hamster cell line HTM18TC8 and of its 2.7 Mb minichromosome derivative retained in the hamster somatic cell hybrid FA3Wg8-4. *(B)* IF-FISH on two representative chromatin fibers from the normal X chromosome showing that hamster Cenp-A (red) was localized to a portion of DXZ1 (green), and asymmetrically distributed. Each fiber shown represents an independent experiment. Arrowed lines denote Cenp-A staining (red). Scale bar is 10 microns. *(C)* IF-FISH on two representative chromatin fibers from the truncated X chromosome showed that CENP-A remained asymmetrically distributed on DXZ1. Each fiber shown represents an independent experiment. Scale bar is 5 microns. *(D)* Genomic sizes of CENP-A binding domains (gray) and DXZ1 arrays (black) on the normal X before and after partial centromere deletion. N = 15 fibers for normal X and n = 20 fibers for deleted X. The asterisk indicates that reduction in CENP-A array size on the deleted X is statisically significant as determined by a Student's t-test (*p* = 0.01).

We next addressed how patterns of chromatin modifications were affected at an X centromere before and after it was structurally altered. The altered X centromere was derived from a single X chromosome that had been transferred into a hamster-human somatic cell hybrid (line HTM18TC8) and systematically truncated to produce deletion derivatives lacking chromosome arms and defined portions of the centromere [16], [21], [22]. These minichromosome derivatives form functional kinetochores, but those lacking over 50% of the centromere and pericentromere are variably stable [22]. The specific derivative X chromosome that we studied (IKNFA3 from line FA3Wg8-4) is 2.7 Mb in size and stable during cell division [22]. This truncated X lacks ∼1 Mb of the original 3 Mb higher-order alpha satellite DXZ1 array and all of the Xq pericentromere (Fig. 7). Thus, the molecular structure of the minichromosome is ∼2 Mb of higher-order alpha satellite DNA DXZ1, 450 kb of Xp pericentromere, and 150 kb of proximal Xp (Fig. 7A). The selectable markers neomycin and hygromycin, used to select for deletion derivatives during chromosomal truncation, flank the derivative X centromere.

First, we tested if the location of Cenp-A was altered by truncation of the X centromere. ChIP with antibodies to hamster Cenp-A were used to optically position Cenp-A on extended chromatin fibers, confirming restriction of Cenp-A to DXZ1 alpha satellite (Fig. 7B,C). On the normal X, Cenp-A spanned 1.12 Mb (±0.38 Mb) of the 3 Mb array of DXZ1 (Fig. 7B, D; n = 15 fibers). We noted that Cenp-A appeared asymmetrically distributed on DXZ1, and was oriented to one end of the fiber (Fig. 7B, note arrowed lines). It was previously reported that centromere proteins are oriented toward the short arm side of DXZ1 [16]. On the truncated version of the same X (deleted X), Cenp-A spanned 0.73 Mb (±0.27 Mb) of the 2 Mb array (Fig. 7C, D; n = 20 fibers). These experiments revealed two intriguing findings. Deleting part of DXZ1 on a human X chromosome in a monochromosomal hybrid reduced the extent of CENP-A assembled on DXZ1. The change in size of the CENP-A domain from 1.12 Mb to 0.73 Mb on the same X chromosome was statistically significant (*p* = 0.01), suggesting that reduction in Cenp-A domain size resulted from structural changes on the X chromosome, presumably at DXZ1 and the Xq pericentromere region. Even though Cenp-A extended over a smaller linear region of DXZ1 on the deleted X, the ratio of CENP-A to DXZ1 remained constant (0.37±0.14) and was not statistically different (*p* = 0.8) between the normal X and deleted X. This result implies that CENP-A is assembled on a similar proportion of DXZ1 HOR alpha satellite irrespective of total array size. This phenomenon has also been observed in a larger study of multiple X and Y centromeres (C. Boivin and B.A. Sullivan, unpublished). We conclude that centromeric chromatin repositions in response to underlying genomic changes at the centromere, but Cenp-A chromatin remains proportional to the total size of the centromeric array.

**Figure 8:**
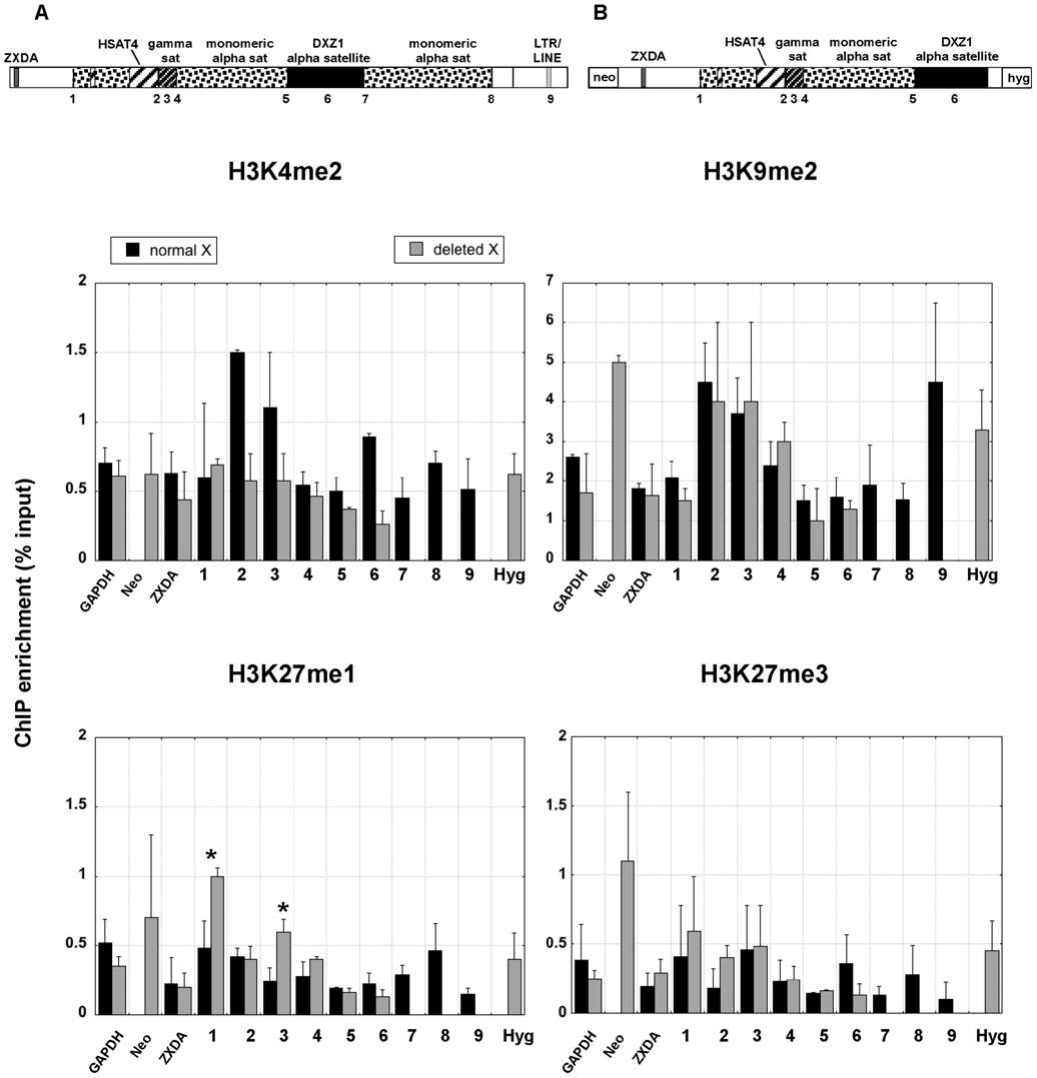
Comparision of histone modifications across a normal and deleted version of the same X centromere. The schematics at the top show the structure of the normal *(A)* and partially deleted *(B)* X centromere. Each chromosome is contained in a human-hamster cell line. The numbers along the centromere represents genomic sites interrogated by ChIP-PCR with a specific histone antibody. The line plots shows average relative enrichment for each histone modification across the centromere region (n = 3 with SD). Control regions, including GAPDH and and X-linked ZXDA are also included. The bar graph shows relative enrichment for H3K27 methylation (n = 3 with SD) calculated as percentage of input. Asterisks indicate statistically significant differences in enrichment for a modification at a particular site between the normal X and deleted X as determined by a Student's t-test (*p*<0.05).

We next asked if the structural rearrangement produced changes in the profile of histone modifications at the deleted X centromere that might explain reduction in Cenp-A domain size (Fig. 8). The distribution of H3K4me2 remained low across the normal X centromere and the deleted X. On the deleted X, the marker genes, neomycin and hygromycin, were as enriched as the X-linked gene ZXDA for H3K4me2 (Fig. 8) and H3K4me3 (data not shown). However, H3K4me2 appeared to be decreased on the deleted X at pericentromeric sites 2 and 3 and at the centromeric site DXZ1. Furthermore, the retained Xp pericentromere showed changes in enrichment for H3K27me1. In particular, it was increased at sites 1 and 3 and slightly decreased at DXZ1 Since the normal and deleted X are genetically identical, we conclude that displacement of pericentric chromatin domains as well as centromeric chromatin domains are a consequence of the large genomic deletion of the X centromere.

## Discussion

In this study, histone modifications correlated with open or repressive chromatin were assayed at intervals spanning the centromeric genome assembly on multiple X chromosomes [12], [13]. Our results provide a broad view of the chromatin assembled within a multi-megabase functional domain on different X chromosomes and in various mammalian backgrounds. Our data confirms that CENP-A is limited to a defined portion of HOR alpha satellite DNA [7], [8]. In each human cell line studied, repressive histone modifications, such as H3K9, H3K27 and H4K20 methylation, were also accumulated at multiple satellite arrays, such as alpha satellite, HSAT4 and gamma satellite. Heterochromatic modifications appeared to decrease beyond the centromere-arm junctions. A similar transition between heterochromatin and euchromatin has been described by a peak of heterochromatic histone modifications on human chromosome 21, implying that other human chromosomes may share similar centromere-arm chromatin boundaries [29].

At some centromeric sites in transformed/immortalized cell lines, H3K4me2 and heterochromatic modifications appeared to coincide. Mammalian tumor cells often exhibit departure from normal patterns of DNA methylation and histone acetylation and methylation, including at repetitive regions [30], [31]. We also observed differences in histone modification patterns (such as H3K4me2 and H3K9me2) between the primary cell line HDF and the transformed cells HT1080 and LT690. These changes may reflect individual differences in chromatin organization or, most likely, consequences of immortalization by viral or oncogenic transformation [32]. In several cell lines, the large arrays of gamma satellite and alpha satellite DNA appeared as a combination of both open chromatin and compact heterochromatin. Chromatin within these satellite regions may be naturally dynamic or the distribution and/or position of the chromatin on the arrays may be functionally significant. Euchromatic and heterochromatic histone modifications mutually reside in other types of repeats such as HOR alpha satellite DNA and centromeric regions in plants and at genes that play key roles in development [8], [9], [28], [33]. A bivalent state may explain the ability of gamma satellite to limit the spread of heterochromatin while simultaneously supporting gene expression [34]. It is presently unclear how heterochromatin and euchromatin are positioned within a given block of gamma satellite. Establishing the orientation and/or distribution of the euchromatin and heterochromatin within the gamma satellite array may reveal further insights into its functional role as a barrier and/or in centromere higher order structure and function.

Somatic cell genetics has a long history in gene identification, mapping and expression, especially in the broader context of metabolic pathways and cancer. Interspecific cell hybrids contain one or more foreign chromosomes that are assembled, replicated, transcribed and segregated by the host's proteins. Although fundamental processes and proteins are similar in mammals, differences in chromosome regulation in a human versus rodent cell have been observed [35]. In the rodent-human cell lines in our study, mouse and hamster Cenp-A were incorporated into the human X centromeres (normal and deleted) contained in each hybrid (Figs. 1, 7). Clearly, an important aspect of centromere function is retained since these chromosomes are mitotically stable. In prior studies of mouse chromosomes, H3K4me2 has been detected at mouse centromeres, but at a much lower level than H3K9 and HK27 methylation and even the histone 2 variant H2A.Z [23], [27], [36], [37]. Our data now adds additional organizational information about a foreign centromere in a rodent background. By ChIP, we also found that H3K4me2 was present at DXZ1 alpha satellite DNA, at or below the level found on the control genes in the two hybrids we analyzed (Figs. 2, 8). These results argue that Cenp-A chromatin in mouse cells may be organized differently than in humans and insects and that when present in hybrid cell lines, human centromere organization reflects that of the mouse host centromere (i.e. more heterochromatic) [23], [27], [37]. Optical mapping of different human chromosomes retained in somatic cell hybrids using chromatin fibers might provide further insights into long-range organization of euchromatic and heterochromatic modifications within human centromeres.

In eukaryotes, the amount of CENP-A chromatin, heterochromatin, and cohesion at the centromere impacts chromosome stability [19], [38], [39]. On normal human chromosomes, only a fraction of alpha satellite is involved in CENP-A chromatin assembly and the remainder of the array is associated with heterochromatin [8], [16]. At the X centromere, centromere proteins such as CENP-C are positioned on the portion of DXZ1 located closest to the X short arm [16] (Fig. 7B). We also saw that Cenp-A was also asymmetrically positioned on a normal X and its deletion derivative (Fig. 7C). The deleted X chromosome was generated by removal of ∼1.0 Mb of the long-arm side of DXZ1 on the parental X. Based on orientation of CENP-A towards the short arm, the deletion should not have truncated the 1.24 Mb Cenp-A domain. Nevertheless, we observed that the Cenp-A domain on the deleted X was reduced to 760 kb (Fig. 7D). There are several possible explanations for this observation. Smaller chromosome size might eliminate the requirement for a larger kinetochore. It has been previously shown using metaphase IF-FISH that X-derived minichromosomes exhibit decreased CENP-A fluorescence with smaller chromosome size [22]. The reduction in CENP-A intensity is supported by and explained by our chromatin fiber analyses showing a contraction of the CENP-A domain. Our ChIP data showed that H3K27me1 increased across the centromere and pericentromere on the deleted X, while other heterochromatic modifications H3K27me3 and H4K20me3 were generally maintained or slightly decreased at the same pericentromeric sites. Interestingly, the 2.7 Mb deleted X minichromosome exhibits reduced stability when transferred from the hamster cell background into different mammalian cells [21], [22]. Chromatin repositioning resulting from contraction of the CENP-A domain in the hamster cells might promote its stability in hamster cells, but compromises centromere function and minichromosome stability in other cell lines due to a smaller kinetochore region. Small chromosome size could limit the degree of chromatin remodeling and/or favor the assembly of heterochromatin, so that the deleted X centromere cannot achieve or maintain appropriate amounts and types of centromeric chromatin. Indeed, it has been reported that increasing heterochromatin on small human artificial chromosomes impairs kinetochore formation and chromosome stability [40]. Our study, combined with other studies in Drosophila, yeast and maize emphasize a similar correlation between the amount of centromeric chromatin and chromosome stability [19], [38], [39].

Our work has extended the profile of histone modifications from the short arm- and long arm-centromere boundaries into the X centromere using sequences that have been excluded from the genomic assembly and from all genomic tiling arrays. As additional genomic information becomes available, finer details of chromatin organization between normal and mutant centromeres can be tested and compared.

## Methods

### Cell culture

Six cell lines were used in this study (Table 1). Human HDF-XSN dermal fibroblasts and HT1080 fibrosarcoma cells were cultured in minimum essential medium alpha (MEM alpha; Invitrogen) supplemented with 10% FBS and antibiotics (Invitrogen). Human lymphoblast cell line LT690 was grown in RPMI supplemented with 15% FBS, antibiotics (Invitrogen) and hypoxanthine-aminopterin-thymidine (HAT; Invitrogen) [15]. Human-mouse somatic cell hybrid lines (L cell derivatives) containing a normal human active X chromosome, AHA-11aB1 [41] and t60-12 [42], and human-Chinese hamster somatic cell hybrid line (HTM18TC8) were grown in MEM-alpha supplemented with HAT (Invitrogen) [43]. A human-Chinese hamster hybrid line (FA3Wg8-4) containing a 2.7 Mb human X minichromosome (IKNFA3) generated by telomere truncation [21] was maintained in MEM-alpha supplemented with antibiotics, 0.5 mg/mL hygromycin B (Invitrogen), and 1 mg/mL G418 sulfate (Mediatech).

### Chromatin immunoprecipitation (ChIP)

Native chromatin containing oligonucleosomes were isolated from cultured cells and prepared by micrococcal nuclease digestion as described [7]. The size of fragmented chromatin ranged between 200–1000 bp. 25 ug of digested chromatin (per IP reaction) was immunoprecipitated with various antibodies as previously described [8]. Antibodies recognizing lysine-specific histone modifications H3K4me2 (ab7766), H3K4me3 (ab8580), H3K9me2 (ab1220 or ab730), H3K9me3 (ab8898), H3K27me1 (Upstate 07-448), H3K27me3 (Upstate 07-449), H4K20me3 (ab9053), and histone variant CENP-A (Abcam ab13939; Millipore 07-754) were purchased from Abcam (Cambridge, MA) and (07-574) Millipore. Anti-mouse Cenp-A and anti-hamster Cenp-A polyclonal antibodies were raised against synthetic Cenp-A N-terminal peptides and produced and purified from rabbit serum by Quality Controlled Biochemicals (QCB, Hopkinton, MA). To control for non-specific binding, a mock control with no antibody was included in each ChIP experiment. One-tenth (2.5 ug) of starting material was kept aside as input DNA control. Chromatin diluted in 1-1.2 ml ChIP Dilution Buffer supplemented with protease inhibitors was pre-cleared with 100 ul salmon sperm DNA/protein A or G agarose slurry for 2 hours at 4°C. Pre-cleared chromatin was incubated with 1–5 ug primary antibodies and 80 ul salmon sperm DNA/protein A or G agarose slurry overnight at 4°C. The precipitated immunocomplexes were washed in a series of buffers (Low Salt Immune Complex Wash Buffer, High Salt Immune Complex Wash Buffer, LiCl Immune Complex Wash Buffer, and 1xTE buffer), and eluted from antibody-bead mixtures with buffer containing 1% SDS and 0.1 M NaHCO_3_. The eluate was treated with proteinase K (40 ug/ml) and RNaseA (40 ug/ml) in 200 mM NaCl, 40 mM Tris-HCl pH 6.5, and 10 mM EDTA pH 8.0 for 1 hour at 42°C. DNA was recovered by phenol/chloroform extraction, and was dissolved in 40 ul of Molecular Biology Grade Water (HyClone). At least three independent ChIP experiments for each antibody were performed.

### PCR Analysis

Immunoprecipitated DNA (IP DNA) was amplified with centromere-specific primers for semi-quantitative PCR using a Biorad iCycler and/or quantitative PCR (QPCR) using a Stratagene MXP3000. Primers were designed using UCSC Genome Browser on March 2006 human reference sequence assembly (NCBI Build 36.1). Primers that amplified higher-order alpha satellite and gamma satellite specific for the human X chromosome were published previously [16], [44]. Primers were validated experimentally as being chromosome X-specific by PCR amplification of DNA from three human male (XY) cell lines and four monochromosomal somatic cell hybrid lines. All primers amplified fragments that were 500 bp or less in size. Primer sequences, UCSC coordinates, and PCR conditions are available upon request. 0.5 ul of IP DNA was used in each PCR reaction, and PCR amplifications were done in duplicate. Semi-quantitative PCR amplifications were subjected to 2% agarose gel electrophoresis and bands were quantified using Image J software (http://rsb.info.nih.gov/). Relative enrichment for histone antibodies at a given genomic position (query) was calculated using the following formula: relative enrichment = [(IP−Mock)/(Input−Mock)]_Query_/[(IP−Mock)/(Input−Mock)]_Normalizer_. Enrichment at centromeric positions was normalized to the enrichment for a STS marker located in euchromatin near ZXDA, the most proximal human gene on Xp. To verify semi-quantitative data for 4 genomic sites, real-time PCRs were done using SYBR Green reporter dye, and analysis and quantification were performed using MxPro^TM^ QPCR Software for Mx3000P^®^ QPCR System (Stratagene) (data not shown). Standard curves, obtained by dilution series of genomic DNA, were used to estimate the relative amounts of DNA correlating relative copy number to treshold cycle (C_t_) values. The dissociation curves with a unique peak verified that PCR products were amplicons of specific regions of interest. Given that mock sample showed no amplification, enrichment was calculated as (IP/Input) _Query_/(IP/Input)_Normalizer_.

### Chromatin fiber immunofluorescence-FISH

Chromatin fibers were obtained from cultured cells according to published protocols [8]. Hamster anti-Cenp-A polyclonal antibodies were incubated at 1∶50 dilution in blocking buffer overnight at 4°C. Anti-rabbit secondary antibodies conjugated to Cy3 (Jackson Immunoresearch) were used to detect the primary antibodies. Slides were washed three times between antibody treatments in 1X PBS+0.05% Tween-20 for 5 minutes at room temperature. Before FISH, slides were fixed in 8% formalin for 5–10 minutes. 5 uL of DXZ1 FISH probe directly labeled with Spectrum Green (Abbott Laboratories) was hybridized to each area of fibers for at least 16 hours at 37°C. Slides were washed in 60–65% formamide/2X SSC, pH 7 at 42°C, followed by several washes in 2X SSC+0.1% Tween-20 at 42°C. Slides were then mounted in Vectashield (Vector Labs, Burlingame, CA) containing 5–10 ug/ml DAPI and visualized on an inverted Olympus IX-71 attached to the Deltavision RT imaging system (Applied Imaging, Inc). Images of chromatin fibers were visualized at 100× magnification. At least 15 individual chromatin fibers were analyzed. Since they often spanned multiple fields of view, they were captured using the “Capture Panels” option in the SoftWoRx application and the fields of view for each fiber were merged together using the “Stitch Image” option. Areas of Cenp-A staining and DXZ1 fluorescence were measured using the “Measure Distances” tool in SoftWoRx. Cenp-A length in megabases was calculated using the formula: Cenp−A_Mb_ = Cenp−A_um_×DXZ1_Mb_/DXZ1_um_. The size of DXZ1 (in megabases) was estimated using restriction digestion and pulsed field gel electrophoresis followed by Southern blotting [16], [21].

### Statistics

Students t-test (two-tailed) was used to calculate significant differences in enrichment between chromatin modifications, chromatin domain sizes, and CENP-A:DXZ1 ratios at centromeres of normal X and deleted X chromosomes. If a *p* value was less than 0.05, it was considered statistically significant.
